# Fraunhofer Diffraction Effects on Total Power for a Planckian Source

**DOI:** 10.6028/jres.106.036

**Published:** 2001-10-01

**Authors:** Eric L. Shirley

**Affiliations:** National Institute of Standards and Technology, Gaithersburg, MD 20899-8441

**Keywords:** diffraction, Fraunhofer, Planckian, power, radiometry

## Abstract

An algorithm for computing diffraction effects on total power in the case of Fraunhofer diffraction by a circular lens or aperture is derived. The result for Fraunhofer diffraction of monochromatic radiation is well known, and this work reports the result for radiation from a Planckian source. The result obtained is valid at all temperatures.

## 1. Introduction

**Fig. 1:**
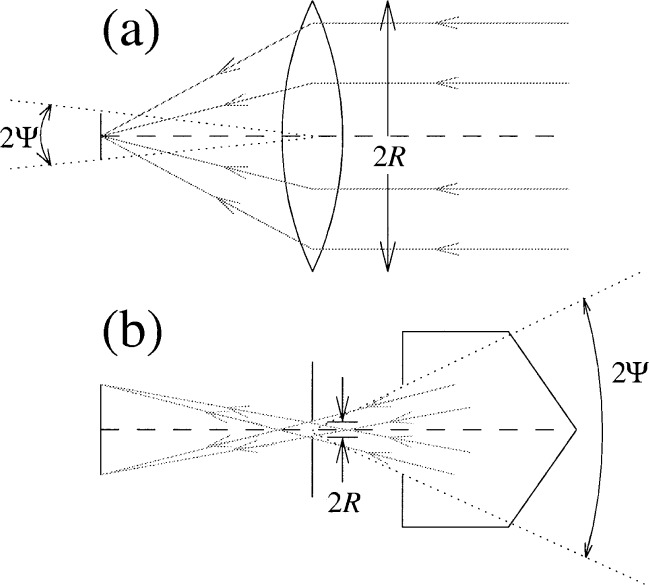
Two optical systems are shown, for which the (spectral) power detected is subject to Fraunhofer diffraction. In the first system (a), a lens concentrates radiation on the detector. In the second system (b), radiation from a blackbody cavity may pass through the blackbody cavity opening, pass through the pinhole aperture, and reach the detector pupil.

Fraunhofer diffraction by a circular lens or aperture is a ubiquitous phenomenon in optics in general and radiometry in particular. Figure 1 illustrates two practical situations in which Fraunhofer diffraction occurs. In the first example, diffraction limits the ability of a lens or other focusing optic to focus light. According to geometrical optics, it is possible to focus rays incident on a lens to converge at a focal point. In practice, even with the focal point at the center of a finite-sized circular detector, some of the light incident on the lens fails to be collected, giving rise to a “diffraction loss.”

The second example can occur when using a source, such as a blackbody cavity source viewed through a small “pinhole” aperture, to calibrate optical systems intended for observing celestial objects or for use in remote-sensing applications. In this case, the angle subtended by the cavity opening at the aperture is larger than the angle subtended by the detector pupil on the dark side of the aperture [1]. Because the former angle is larger than the latter one, geometrical optics suggests that the total power detected depends only on the black-body temperature and a geometrical factor related to the pinhole aperture, detector optics, and relative separation, because the detector pupil is overfilled. However, diffraction leads to losses in the total power reaching the detector.

All of the above diffraction losses have been a subject of considerable interest, and they have been considered by Blevin [2], Boivin [3], Steel, De, and Bell [4], and Shirley [5]. The formula for the relative diffraction loss in spectral power is already well known, and it is noted below. However, a formula for the diffraction loss in the total power for the case of a Planckian source such as a star or blackbody appears to have been given only in the high-temperature limit. This article reports a formula for the diffraction loss in the power for such a source at all temperatures. The formula is useful for predicting the loss in cases such as the examples discussed, and it can be used as an independent check for numerically calculated diffraction losses.

For the radiation present at a given wavelength λ, the diffraction loss can be described in terms of a unit-less parameter, *v* = 2π*ψR*/*λ*. *R* is the radius of the lens or aperture. The angle *ψ* is defined in either of two ways: either 2*ψ* is the full angle subtended by the detection pupil at the focusing optic, or 2*ψ* is the full angle subtended by the blackbody cavity opening at the pinhole aperture. For a source in thermal equilibrium at temperature *T*, the effects of temperature on the distribution of spectral power enter relevant equations through the ratio, *c*_2_/(*λT*) ≡ *Av*. Here, *c*_2_ = *hc*/*k* = 1.438 7752(25) × 10^−2^ m K is the second radiation constant, where the number in parenthesis is the one-standard-deviation uncertainty in the last two digits. This implicitly defines another unit-less parameter, *A* = *c*_2_/(2π*ψRT*). The relative effects of diffraction on the total power only depend on this one parameter, *A*.

As a final note, if the detection pupil subtends a considerable angle, the diffraction losses can be approximately given as the weighted average of losses arising, in the case of an infinitesimal detector, for values of *ψ* varying between *ψ* minus the half-angle subtended by the detector and *ψ* plus the same half-angle. Analogous weighting can be used, in the context of the first example, to account for the finite angular diameter of a source (smaller than 2*ψ*). Such averaging is discussed elsewhere [5] but is not discussed further in this work, which henceforth assumes a single effective value of *ψ*.

## 2. Derivation of Formula

In the Fraunhofer case, the ratio of the true spectral power to the spectral power predicted by geometrical optics is

$$F\left(v\right)=1-J_{0}^{2}\left(v\right)-J_{1}^{2}\left(v\right).$$ (1)

Here, J*_m_*(*v*) is a cylindrical Bessel function. It is necessary to incorporate this wavelength-dependent factor when evaluating the ratio of the total power detected to the total power predicted by geometrical optics. For a source whose spectral output obeys the Planck distribution, this unit-less ratio is given by

$$\bar{F}\left(A\right)=\frac{\int_{0}^{\infty }dvv^{3}{\left[\exp\left(Av\right)-1\right]}^{-1}\left[1-J_{0}^{2}\left(v\right)-J_{1}^{2}\left(v\right)\right]}{\int_{0}^{\infty }dvv^{3}{\left[\exp\left(Av\right)-1\right]}^{-1}}.$$ (2)

The value of the denominator is familiar, being given by

$$\begin{array}{c} \int_{0}^{\infty }dvv^{3}{\left[\exp\left(Av\right)-1\right]}^{-1}=\sum_{n=1}^{\infty }\int_{0}^{\infty }dvv^{3}\exp\left(-nAv\right)\\ =6\sum_{n=1}^{\infty }{\left(nA\right)}^{-4}=6A^{-4}\zeta \left(4\right). \end{array}$$ (3)

Here, *ζ*(*n*) is the Riemann zeta function. When evaluating the numerator, two techniques have been found to be helpful: one for the “low-temperature” case (defined as *A* > 4) and one for the “high-temperature” case (defined as *A* < 4). By such definitions, “high” and “low” temperature cases arise depending on the average diffraction loss for a source at temperature *T*. This depends on *T* and the geometry through *A*. In either case, one first makes use of the relation,

$$J_{0}\left[2v\;\sin\left(\theta /2\right)\right]=J_{0}^{2}\left(v\right)+2\sum_{m=1}^{\infty }J_{m}^{2}\left(v\right)\cos\left(m\theta \right),$$ (4)

from which follows,

$$J_{0}^{2}\left(v\right){\text{+J}}_{1}^{2}\left(v\right)=\frac{1}{2\pi }\int_{0}^{2\pi }d\theta \left(1+\cos\theta \right)J_{0}\left[2v\;\sin\left(\theta /2\right)\right].$$ (5)

### Low-Temperature Case

To evaluate the numerator in the low-temperature case, series expansion of the latter Bessel function in Eq. (5) yields

$$\begin{array}{l} J_{0}^{2}\left(v\right){\text{+J}}_{1}^{2}\left(v\right)=\frac{1}{\pi }\sum_{s=0}^{\infty }\frac{{\left(-1\right)}^{s}v^{2s}}{{\left(s!\right)}^{2}}\int_{0}^{2\pi }d\theta \left[1-\sin^{2}\left(\theta /2\right)\right]\sin^{2s}\left(\theta /2\right)\\ =2\sum_{s=0}^{\infty }\frac{{\left(-1\right)}^{s}v^{2s}}{{\left(s!\right)}^{2}}\left[\frac{(2s-1)!!}{(2s)!!}-\frac{(2s+1)!!}{(2s+2)!!}\right]\\ =2\sum_{s=0}^{\infty }\frac{{\left(-1\right)}^{s}v^{2s}}{{\left(s!\right)}^{2}}\left[\frac{(2s-1)!!}{(2s+2)!!}\right] \end{array}$$ (6)

This permits one to rewrite Eq. (2) as follows:

$$\begin{array}{l} Q(A)\equiv 6A^{-4}\zeta (4)[1-\bar{F}(A)]\\ =\sum_{n=1}^{\infty }\int_{0}^{\infty }dvv^{3}\exp(-nAv)[J_{0}^{2}(v)+J_{1}^{2}(v)]\\ =2\sum_{s=0}^{\infty }\frac{{\left(-1\right)}^{s}(2s+3)!\zeta (2s+4)}{{\left(s!\right)}^{2}A^{2s+4}}\left[\frac{(2s-1)!!}{(2s+2)!!}\right]\\ =-\sum_{s=0}^{\infty }\frac{{\left(2\pi \right)}^{2s+4}(2s+3)!(2s-1)!!B_{2s+4}}{{\left(s!\right)}^{2}(2s+4)!(2s+2)!!A^{2s+4}}, \end{array}$$ (7)

In this expression, *B_i_* is a Bernoulli number. The sum converges for all *A* > 2, and one obtains an error in $\bar{F}\left(A\right)$ estimated to be as small as 10^−14^ for *A* > 4 if one sums up to *s* = 28.^6^

### High-Temperature Case

To evaluate the numerator in the high-temperature case, the relation in Eq. (2) is rewritten to render

$$\begin{array}{l} Q(A)=6A^{-4}\zeta (4)[1-\bar{F}(A)]\\ =\sum_{n=1}^{\infty }\int_{0}^{\infty }dvv^{3}\exp(-nAv)[J_{0}^{2}(v)+J_{1}^{2}(v)]\\ =\sum_{n=1}^{\infty }Q_{1}(nA), \end{array}$$ (8)

with

$$Q_{1}(A)=\int_{0}^{\infty }dvv^{3}\exp(-Av)[J_{0}^{2}(v)+J_{1}^{2}(v)].$$ (9)

Use of Eq. (5) and the relation,

$$\int_{0}^{\infty }dv\exp(-\alpha v)J_{0}(\beta v)=\frac{1}{\sqrt{\alpha^{2}+\beta^{2}}},$$ (10)

one has

$$Q_{1}(A)={\left(-\frac{d}{dA}\right)}^{3}\frac{1}{2\pi }\int_{0}^{2\pi }d\theta \frac{1+\cos\theta }{\sqrt{A^{2}+4\sin^{2}(\theta /2)}}.$$ (11)

Making the abbreviation, *w* = 4/(*A*^2^ + 4), one has

$$\begin{array}{l} Q_{1}(A)={\left(-\frac{d}{dA}\right)}^{3}\left[\frac{4}{\pi \sqrt{A^{2}+4}}\int_{0}^{\pi /2}d\phi \frac{\cos^{2}\phi }{\sqrt{1-w\cos^{2}\phi }}\right]\\ ={\left(-\frac{d}{dA}\right)}^{3}\left\{\frac{4}{\pi \sqrt{A^{2}+4}}\left[\frac{K(w)-E(w)}{w}\right]\right\}\\ ={\left(-\frac{d}{dA}\right)}^{3}\left\{\frac{\sqrt{A^{2}+4}}{\pi }[K(w)-E(w)]\right\}, \end{array}$$ (12)

where *E*(*w*) and *K*(*w*) are respectively complete elliptic integrals of the first and second kind, defined according to the convention,^7^

$$\begin{array}{l} E(w)=\int_{0}^{\pi /2}d\phi \sqrt{1-w\sin^{2}\phi },\\ K(w)=\int_{0}^{\pi /2}\frac{d\phi }{\sqrt{1-w\sin^{2}\phi }}. \end{array}$$ (13)

(In a different convention, *w* is replaced by *w*^2^ in the integrand but nowhere else.) From the properties of elliptic integrals [8], one may obtain

$$Q_{1}(A)=\frac{4}{\pi A^{3}}\left[\frac{4(2+A^{2})E(w)-A^{2}K(w)}{{\left(A^{2}+4\right)}^{3/2}}\right],$$ (14)

and

$$Q_{1}(A)=-\frac{d}{dA}\left[\frac{4E(w)}{\pi A^{2}\sqrt{A^{2}+4}}\right].$$ (15)

The latter result is easily obtained, because *Q*_1_(*A*) has already been written as the third derivative of an expression with respect to *A*.

The closed-form result in Eq. (14) may be directly substituted in Eq. (8), after which one may sum over *n*. Because the summand can be integrated according to Eq. (15), however, the Euler-Maclaurin formula may be used to obtain a more easily evaluated, asymptotic expression for *Q*(*A*):

$$\begin{array}{l} Q(A)=\frac{4\zeta (3)}{\pi A^{3}}+\sum_{n=1}^{N-1}{\tilde{Q}}_{1}(nA)+\frac{1}{A}\int_{NA}^{\infty }dA^{\prime }{\tilde{Q}}_{1}(A^{\prime })+\frac{1}{2}{\tilde{Q}}_{1}(NA)\\ -{\left[\sum_{s=1}^{m-1}\frac{B_{2s}}{(2s)!}A^{2s-1}{\left(\frac{d}{dA^{\prime }}\right)}^{2s-1}{\tilde{Q}}_{1}(A^{\prime })\right]|}_{A^{\prime }=NA}+R_{m}(N,A), \end{array}$$ (16)

where *B_i_* is again a Bernoulli number. Here a new function,

$${\tilde{Q}}_{1}(A)=Q_{1}(A)-\frac{4}{\pi A^{3}},$$ (17)

has been introduced as the summand, so that the largest part of *Q*_1_(*A*) is extracted from the sum prior to summation.

The parameters *m* and *N* are both positive integers. *R_m_*(*N*, *A*), the remainder (i.e., error) term, usually decreases initially with increasing *m*, but then rapidly diverges. It is therefore good to choose a suitable combination of *N* and *m*, for a given value of *A*, to have an efficient yet accurate result. Increasing *N* should usually improve results, and one obtains an error in $\bar{F}\left(A\right)$ estimated to be as small as 10^−14^ for *A* < 4 if one uses *N* = 60 and *m* = 5.

Explicitly, *Q*(*A*) is given by

$$\begin{array}{l} Q(A)=\frac{4\zeta (3)}{\pi A^{3}}+\sum_{n=1}^{N-1}{\tilde{Q}}_{1}(nA)\\ +\frac{2}{\pi \alpha^{3}}\left\{\frac{\left[8(N+1)+2\alpha^{2}(N+2)]E(\omega )-\alpha^{2}K(\omega \right)}{{\left(4+\alpha^{2}\right)}^{3/2}}-N-1\right\}\\ +\frac{1}{\pi \alpha^{3}}\left(-\frac{1}{N}+\frac{1}{3N^{3}}-\frac{1}{3N^{5}}+\frac{3}{5N^{7}}-\cdots \right)\\ +\frac{1}{\pi \alpha^{3}}\sum_{s=1}^{m-1}\frac{B_{2s}}{(2s)!}\frac{[e_{s}(\alpha )E(\omega )-\alpha^{2}k_{s}(\alpha )K(\omega )]f_{s}}{N^{2s-1}{\left(4+\alpha^{2}\right)}^{(4s+1)/2}}\\ +R_{m}(N,A). \end{array}$$ (18)

One should include only the first *m −* 1 terms in the parenthetic expression preceding the second sum. Two abbreviations, *α* = *NA* and *ω* = 4/(4 + *α*^2^), have been introduced. In the second sum, the first several constants are given by

$$\begin{array}{l} \frac{B_{2}}{2!}=\frac{1}{12},\;\frac{B_{4}}{4!}=-\frac{1}{720},\;\frac{B_{6}}{6!}=\frac{1}{30240},\;\frac{B_{8}}{8!}=-\frac{1}{1209600},\\ f_{1}=4,\;f_{2}=24,\;f_{3}=48,\;f_{4}=2880. \end{array}$$ (19)

Likewise, the first several *e*- and *k*-functions are given by

$$\begin{array}{l} e_{1}(\alpha )=96+68\alpha^{2}+19\alpha^{4},\\ k_{1}(\alpha )=12+7\alpha^{2},\\ e_{2}(\alpha )=5120+6112\alpha^{2}+2880\alpha^{4}+630\alpha^{6}+117\alpha^{8},\\ k_{2}(\alpha )=640+624\alpha^{2}+216\alpha^{4}+57\alpha^{6},\\ e_{3}(\alpha )=1720320+2908160\alpha^{2}+2093376\alpha^{4}+829552\alpha^{6}+196764\alpha^{8}+19917\alpha^{10}+3726\alpha^{12},\\ k_{3}(\alpha )=215040+316480\alpha^{2}+193072\alpha^{4}+62892\alpha^{6}+8265\alpha^{8}+2046\alpha^{10},\\ e_{4}(\alpha )=33030144+72310784\alpha^{2}+70087936\alpha^{4}+39429888\alpha^{6}+14163680\alpha^{8}+3350128\alpha^{10}+572853\alpha^{12}+21062\alpha^{14}+6121\alpha^{16},\\ k_{4}(\alpha )=4128768+8135680\alpha^{2}+6993408\alpha^{4}+3421440\alpha^{6}+1037920\alpha^{8}+219516\alpha^{10}+8820\alpha^{12}+3601\alpha^{14}. \end{array}$$ (20)

## 3. Evaluation of Formula

In the high-temperature limit, this gives a result of the form

$$\bar{F}(A)=1-\frac{\left[4A\zeta (3)/\pi +O(A^{3}\;\ln\;A)\right]}{6\zeta (4)},$$ (21)

and evaluating the successive terms in Eq. (21) may be both difficult and unfruitful, because the series appears to be both very complicated and slow to converge. Note that the leading terms in Eq. (21) are consistent with other works cited, such as the work of Blevin. In the low-temperature limit, one has

$$\bar{F}(A)=\frac{10\pi^{2}}{21A^{2}}-\frac{\pi^{4}}{4A^{4}}+\cdots .$$ (22)

**Fig. 2:**
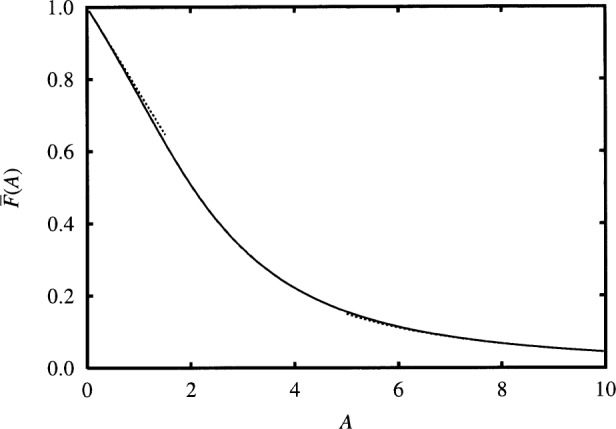
$\bar{F}\left(A\right)$ vs *A* according to Eq. (7) and Eq. (18) (solid line), and Eqs. (21–22) (dotted line). The latter, approximate results apply in the limits of small *A* and large *A*.

Figure 2 shows $\bar{F}\left(A\right)$ for a range of values of *A* bridging the low- and high-temperature regions. The solid curve indicates “exact” results obtained using Eq. (7) for *A* > 4 and Eq. (18) for *A* < 4. The dashed curves indicate approximate results for low- and high-temperature limits, obtained using the respective formulas in Eqs. (21–22). Likewise, Table 1 shows sample values of $\bar{F}\left(A\right)$ over a similar range. For 0 < *A* < 0.2 and *A* > 8, inclusion of terms shown in Eqs. (21–22) yields an error in $\bar{F}\left(A\right)$ smaller than 0.001. While this immediate discussion helps provide a sense of the behavior of $\bar{F}\left(A\right)$, accurate values should be found using Eq. (7) and Eq. (18).

## Figures and Tables

**Table 1:** $\bar{F}\left(A\right)$ at sample values of *A*

*A*	$\bar{F}\left(A\right)$	*A*	$\bar{F}\left(A\right)$	*A*	$\bar{F}\left(A\right)$	*A*	$\bar{F}\left(A\right)$
0.2	0.9526	2.2	0.4643	4.2	0.2055	6.2	0.1077
0.4	0.9041	2.4	0.4259	4.4	0.1912	6.4	0.1018
0.6	0.8543	2.6	0.3907	4.6	0.1781	6.6	0.0964
0.8	0.8033	2.8	0.3587	4.8	0.1663	6.8	0.0914
1.0	0.7514	3.0	0.3297	5.0	0.1555	7.0	0.0867
1.2	0.6994	3.2	0.3034	5.2	0.1457	7.2	0.0824
1.4	0.6481	3.4	0.2797	5.4	0.1367	7.4	0.0784
1.6	0.5984	3.6	0.2582	5.6	0.1285	7.6	0.0746
1.8	0.5508	3.8	0.2389	5.8	0.1210	7.8	0.0712
2.0	0.5060	4.0	0.2214	6.0	0.1141	8.0	0.0679
